# Supplementary material for: There You Are! Automated Detection of Indris’ Songs on Features Extracted from Passive Acoustic Recordings
Source: Animals (Basel). 2023 Jan 9;13(2):241. doi: 10.3390/ani13020241 (PMC9855168; doi:10.3390/ani13020241)
Supplement: Supplementary file 1 [file animals-13-00241-s001.zip › animals-2116428-Supplementary Materials.pdf]

Supplementary Materials

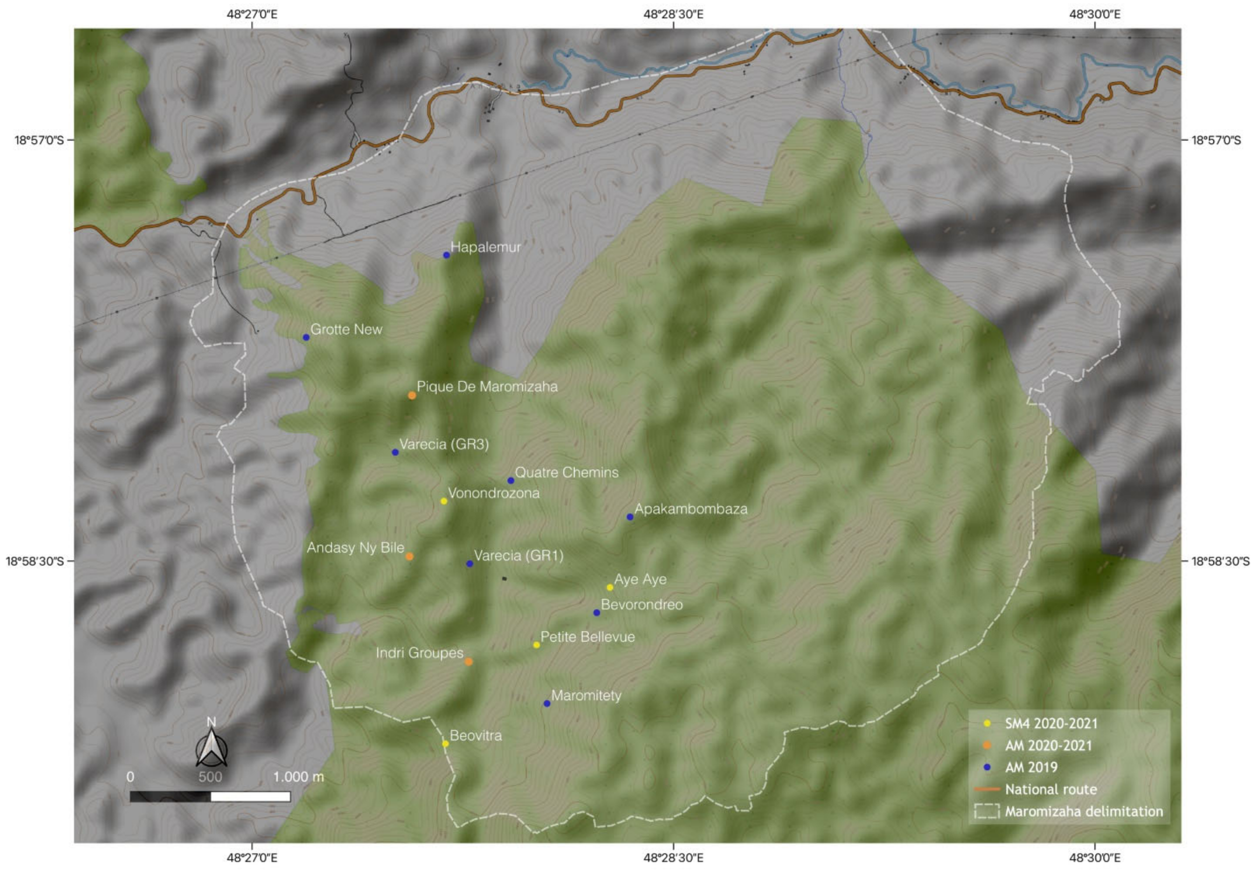

Figure S1: The coordinates of the sites chosen for the recordings of the three datasets are plotted on the map of the Maromizaha New Protected Area; the forested area is shaded in green.

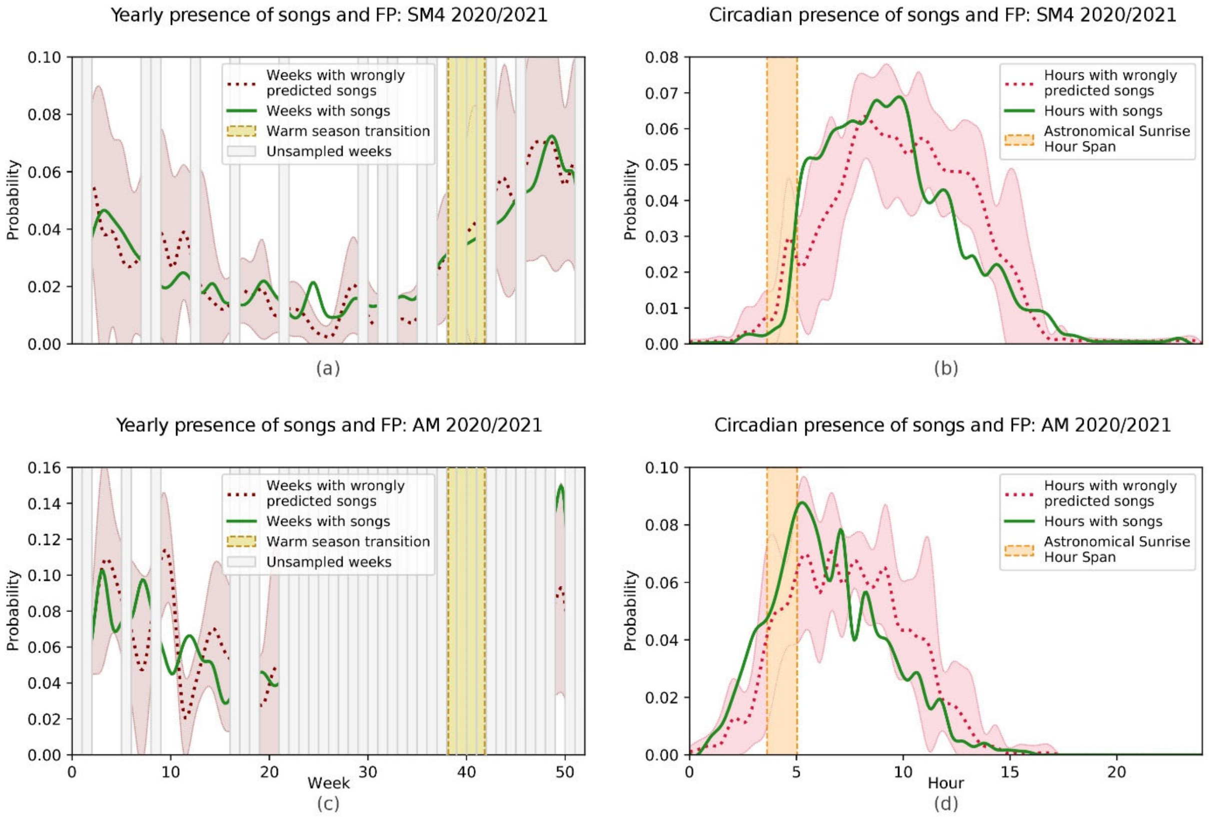

Figure S2: Yearly and circadian trends of the presence of songs and songs' predictions that did not contain any song (False Positives). **a)** and **b)** report data for the SM4 2020/2021 dataset; **c)** and **d)** report data for the AM 2020/2021 dataset. We considered a week unsampled if less than 250 recordings (≈ 40 hours) were present; we did not use recordings of unsampled weeks to compute the probabilities for plots **a)** and **c)**, related to yearly trends, while we did employ all recordings for plots **b)** and **d)**, related to circadian trends. In the plots representing the yearly trend, we highlighted the typical period that presents a transition between the cold and the warm season [35], while for those concerning the circadian trends, we highlighted the astronomical sunrise hour span [54]. Dotted lines and shaded areas represent respectively the mean and the area comprise between three standard deviations obtained from the ten iterations of cross-validation. We reported the trends regarding Erroneous and Missed Predictions in Figure S2 and Figure S3, respectively.

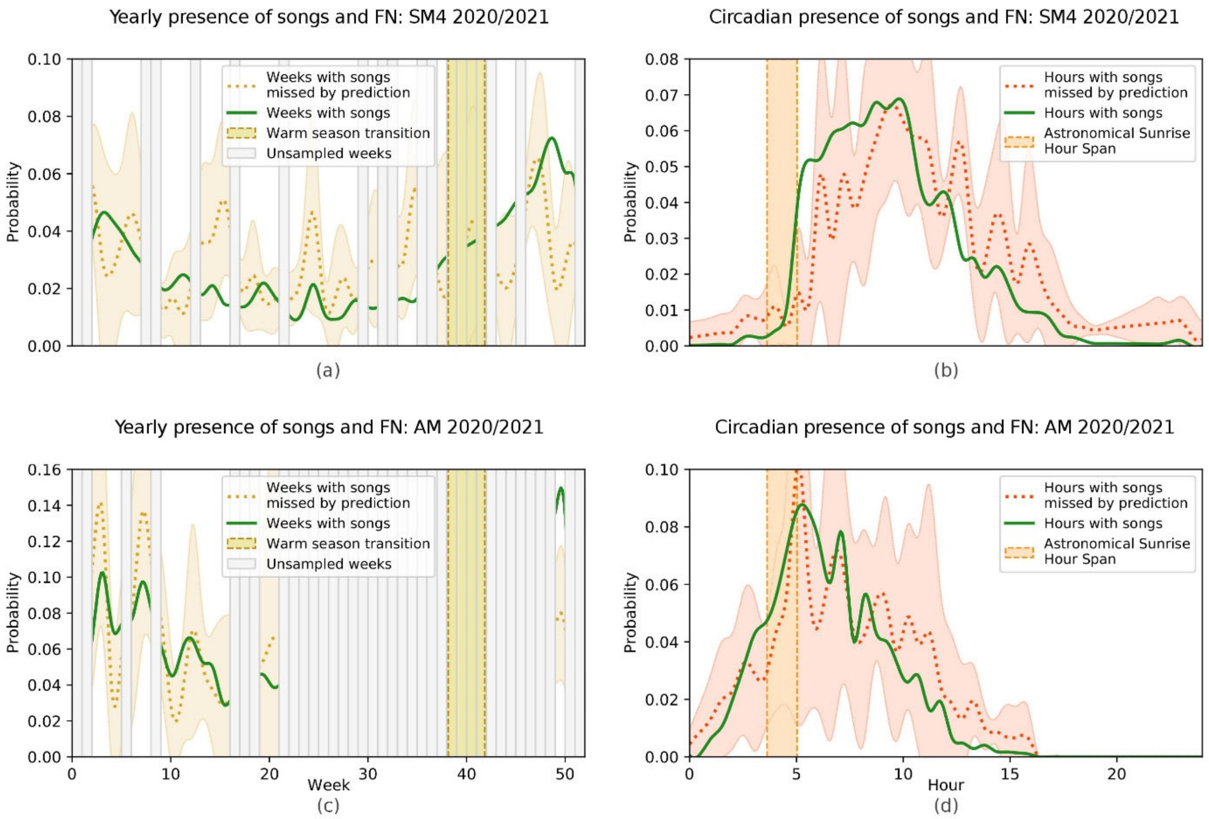

Figure S3: Yearly and circadian trends of the presence of songs and songs' which were not correctly classified by the prediction (False Negatives). **a)** and **b)** report data for the SM4 2020/2021 dataset; **c)** and **d)** report data for the AM 2020/2021 dataset. We considered a week unsampled if less than 250 recordings (≈ 40 hours) were present; we did not use recordings of unsampled weeks to compute the probabilities for plots **a)** and **c)**, related to yearly trends, while we did employ all recordings for plots **b)** and **d)**, related to circadian trends. In the plots representing the yearly trend, we highlighted the typical period that presents a transition between the cold and the warm season [35], while for those concerning the circadian trends, we highlighted the astronomical sunrise hour span [54]. Dotted lines and shaded areas represent respectively the mean and the area comprise between three standard deviations obtained from the ten iterations of cross-validation. We reported the trends regarding Erroneous and Missed Predictions in Figure S2 and Figure S3, respectively.

Table S1. The name of the sites and the label we used to display them in Figure 2; for each site, we reported the coordinates, average number of recordings, average song occurrence, and average recall; we highlighted the average number of recordings when >250, i.e. the threshold we chose to display them on the graph. We reported data relative to the three test sets.

Site	Label	Coordinates	Average number of recordings	Average song occurrence	Average recall
			**AM 2019**		
Hapalemur	a	18°57 24'' S, 48°27'41'' E	**451**	0.06	0.69
Vonondrozona	b	18°58'17'' S, 48°27'40'' E	**527**	0.16	0.9

Quatres Chemins	c	18°58'12'' S, 48°27'55'' E	**355**	0.11	0.86
Aye Aye	d	18°58'35'' S, 48°28'16'' E	**268**	0.12	0.86
Beovitra	e	18°59'08'' S, 48°27'41'' E	**434**	0.14	0.79
Petite Bellevue		18°58'47'' S, 48°28'00'' E	159	0.06	0.81
Apakambombaza		18°58'20'' S, 48°28'20'' E	74	0.16	0.64
Varecia (GR1)		18°58'30'' S, 48°27'46'' E	41	0.29	0.84
Varecia (GR3)		18°58'06'' S, 48°27'30'' E	101	0.10	0.09
Bevorondreo		18°58'41'' S, 48°28'13'' E	97	0.14	0.89
Grotte New		18°57'42'' S, 48°27'11'' E	171	0.23	0.79
Maromitety		18°59'00'' S, 48°28'02'' E	133	0.29	0.81
**AM 2020/2021**					
Pique De Maromizaha	f	18°57'54'' S, 48°27'34'' E	**1981**	0.08	0.89
Andasy Ny Bile	g	18°58'29'' S, 48°27'33'' E	**2088**	0.12	0.90
Indri Groupes	h	18°58'51'' S, 48°27'46'' E	**2069**	0.10	0.80
Bevorondreo	i	18°58'41'' S, 48°28'13'' E	**2191**	0.06	0.85
Maromitety	j	18°59'00'' S, 48°28'02'' E	**1692**	0.11	0.91
Beovitra		18°59'08'' S, 48°27'41'' E	108	0.20	0.92
**SM4 2020/2021**					
Vonondrozona	k	18°58'17'' S, 48°27'40'' E	**3790**	0.10	0.92
Aye Aye	l	18°58'35'' S, 48°28'16'' E	**6728**	0.09	0.72
Petite Bellevue	m	18°58'47'' S, 48°28'00'' E	**3769**	0.12	0.82
Beovitra	n	18°59'08'' S, 48°27'41'' E	**6089**	0.09	0.92
