# Supplementary figures and images for: There You Are! Automated Detection of Indris’ Songs on Features Extracted from Passive Acoustic Recordings
Source: Animals (Basel). 2023 Jan 9;13(2):241. doi: 10.3390/ani13020241 (PMC9855168; doi:10.3390/ani13020241)

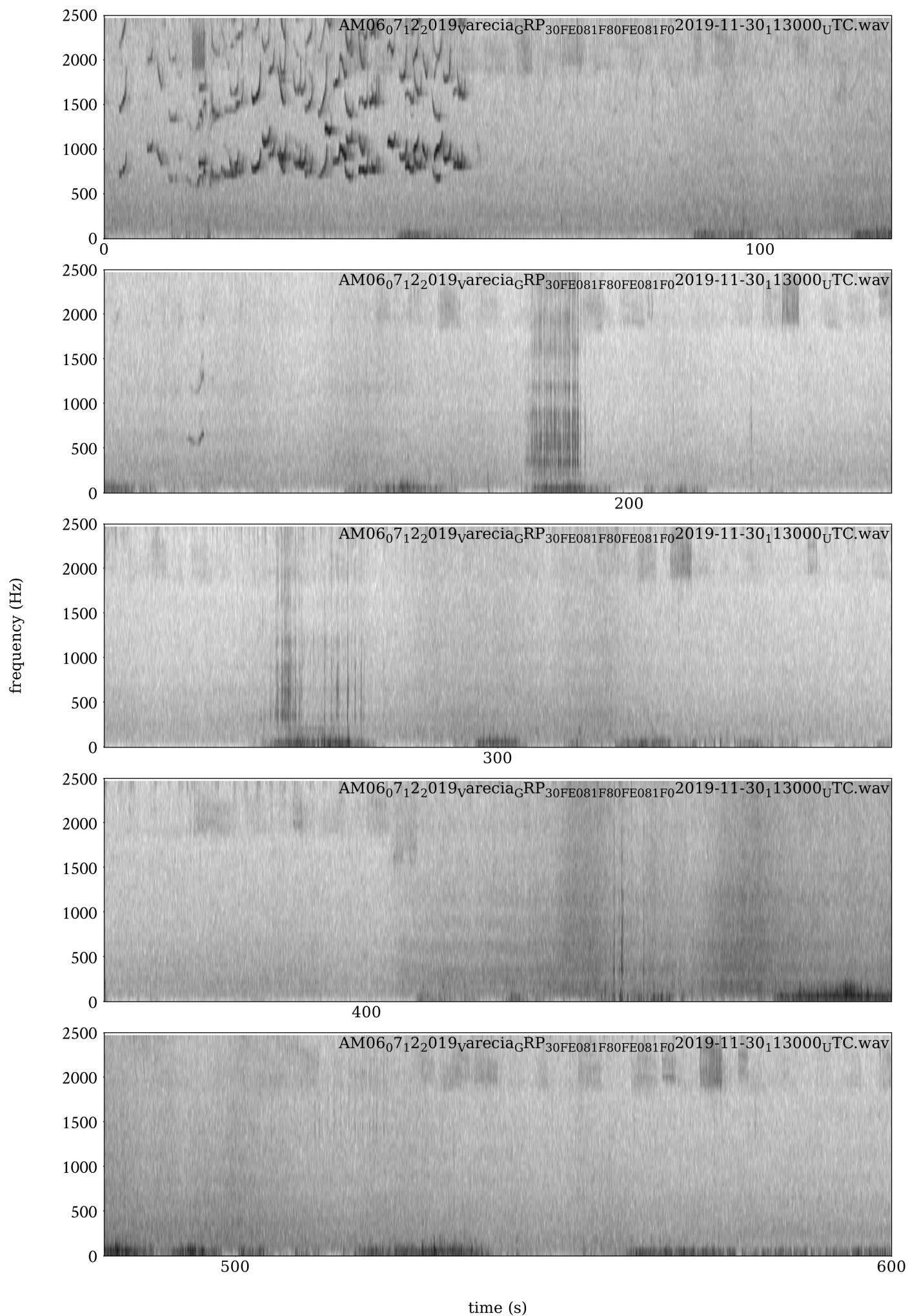

Supplement: Supplementary file 1 [file animals-13-00241-s001.zip › Sample_spectrogram.pdf]
